# Accelerating Fish Sauce Fermentation: Integrating Microbial, Biochemical and Physical Transformations for Optimised Processing

**DOI:** 10.17113/ftb.64.03.26.9498

**Published:** 2026-09-15

**Authors:** Shanti Dwita Lestari, Norhayati Hussain, Honey Yahdillah, Nasiru Bilkisu Umar, Afiah Mohamed Ghazali

**Affiliations:** 1 Faculty of Food Science and Technology, Universiti Putra Malaysia; 2 Fisheries Product Technology Department, Universitas Sriwijaya, Indonesia

**Keywords:** backslopping, autolysis, proteolytic activity, microbial community analysis

## Abstract

Fish sauce is a traditional fermented condiment produced through prolonged fermentation of fish and salt, resulting in a complex flavour profile driven by microbial and biochemical transformations. However, conventional processing requires extended fermentation periods, limiting production efficiency and consistency. High salt concentrations create a selective environment that suppresses undesirable microorganisms while favouring halotolerant communities that drive proteolysis and flavour development. This review synthesises current advances in fish sauce fermentation by integrating microbiological, biochemical and physical perspectives. Emphasis is placed on microbial community dynamics revealed through both culture-dependent and culture-independent approaches, and on their roles in protein hydrolysis and metabolite formation. The contributions of key microbial groups, including *Bacillus*, *Tetragenococcus* and *Virgibacillus*, to proteolytic activity and flavour generation are critically evaluated. Emerging strategies to accelerate fermentation, like starter culture application and backslopping, are assessed in the context of microbial functionality and process control. Evidence suggests that targeted manipulation of microbial consortia can enhance enzymatic activity, shorten fermentation time, and improve product consistency. This review provides a framework for optimising fish sauce fermentation by linking microbial ecology with biochemical transformations and processing strategies. Key knowledge gaps are identified, particularly in the standardisation of microbial consortia and the integration of omics-driven approaches for controlled fermentation design.

## INTRODUCTION

Fish sauce is a liquid condiment produced by fermenting fish with high contents of salt, typically containing more than 20 % sodium chloride and fermented at 30–35 °C for at least six months (*1*). Across Asia, fish sauce is known by various names, including *budu* (Malaysia), *nam pla* (Thailand) and *nuoc mam* (Vietnam). Its distinctive savoury flavour profile makes fish sauce a widely used seasoning and culinary ingredient in many Asian cuisines. In recent years, increasing consumer demand for convenient, nutritious, flavour-rich, low-sodium seasoning options, combined with the global popularity of Asian food, has driven steady growth of the fish sauce market. The global market is projected to expand from USD 15.38 billion in 2021 to USD 20.79 billion by 2029 (*2*). Thailand and Vietnam remain the world’s largest producers, with Vietnam recording approx. 417.84 million litres of fish sauce in 2021; an increase of 42.42 million litres from the previous year (*2*). Despite its long history and widespread consumption across Asia, conventional fish sauce fermentation remains time-consuming, often requiring several months to more than a year to develop the desired flavour, aroma and chemical maturity. This extended fermentation period, together with differences in raw materials, fish-to-salt ratios, microbial ecology, processing conditions and regional practices, contributes to substantial variability in product quality, safety and sensory characteristics.

Fish sauce production is regulated differently across countries, and global practices remain diverse and non-standardised (*3*). The most widely recognised global reference is the Codex Standard for Fish Sauce established by the Food and Agriculture Organization of the United Nations/World Health Organization (FAO/WHO) Codex Alimentarius Commission (*4*). The standard defines fish sauce as a translucent, non-turbid liquid with a salty taste and characteristic fish flavour, obtained from fermentation of fish and salt for a minimum of six months. Codex also specifies permitted additives, including acidity regulators (tartrates, citrates, malate, ascorbic acid, sodium lactate and acetic acid), flavour enhancers (monosodium glutamate, inosinic acid, disodium inosinate and disodium guanylate) and preservatives (benzoates and sorbates, up to 1000 mg/kg). Caramel III is the only allowable colourant, permitted up to 50 000 mg/kg. Products containing sediments (other than NaCl crystals) or exhibiting cloudiness are considered defective. Additional regulations exist at the national level, such as Malaysia’s Food Regulations 1985 (*5*) for budu and Thailand’s Notification of the Ministry of Public Health No. 203 Buddhist Era (B.E.) 2543 (*6*). The quality parameters for fish sauce specified in selected national and international regulations are summarised in Table 1 (*4*-*7*).

**Table 1:** Quality parameters of fish sauce listed in regulations from different countries

Quality parameter	*Budu* (Malaysian Food Regulation 1985, 2017) (*5*)	*Nam pla* (Thai Public Health Notification No. 203 B.E. 2543, 2000) (*6*)	*Nuoc mam* (Vietnamese standard TCVN 5107:2003) (*7*)	Fish sauce (standard CXS 302-2011) (*4*)
*γ*(NaCl)/(g/L)	min 15*	min 200	200–285	min 200
*w*(protein)/%	min 5	nd	nd	nd
*γ*(TN)/(g/L)	nd	min 9	≥10 (minimum for "grade 2")	min 10
(*m*(AN)/*m*(TN))/%	nd	40–50	≥40	min 40
(*m*(glutamic acid)/*m*(TN))/%	nd	0.4–0.6	nd	nd
pH	nd	nd	4.5–6.5	5.0–6.5
*γ*(natural sediment)/(g/L)	nd	max 0.1	not allowed	nd

*Expressed in %; nd=no data specified in the specific document, TN=total nitrogen, AN=amino nitrogen

Recent comparative studies have highlighted significant differences in chemical composition and food safety parameters among commercial fish sauces across Asian countries, reflecting variability in raw materials, processing methods and regulatory frameworks (*8*). These findings highlight the need for more systematic process optimisation, particularly when accelerated fermentation strategies are introduced, as such interventions may alter microbial succession, biochemical reactions, flavour formation and safety-related compounds.

Fish sauce production commonly uses small pelagic fish species such as anchovies (*Stolephorus* sp. and *Engraulis* sp.), sardines (*Sardinella* sp.), herrings (*Clupea* sp.), mackerel (*Rastrelliger* sp., *Decapterus* sp. and *Scomber scombrus*), *Gambusia affinis* and Pacific whiting (*Merluccius* sp.) (*9*). Compared to mammalian meat, fish muscle contains less connective tissue and has shorter fibres, facilitating rapid enzymatic breakdown (*10*). During fermentation, protein breakdown is driven by endogenous fish enzymes, microbial enzymes and salt-mediated physicochemical changes. *Post-mortem* autolysis releases cathepsins, proteases and other hydrolases, which soften muscle tissue and generate peptides, free amino acids and other soluble nitrogenous compounds (*11*, *12*). These biochemical transformations are central to liquefaction, umami development, aroma generation and overall product maturation.

Fish sauces vary widely in chemical composition depending on raw materials, geographic origin, fermentation conditions and processing methods. The pH generally ranges from 5.0 to 6.5, with acetic acid as the dominant organic acid (*13*, *14*). Commercial salt typically ranges from 20 to 30 %, although some traditional products like Iranian *mahyaveh*, Indonesian *bakasang* and Japanese *shottsuru* contain <20 % NaCl (*14*–*17*). Lipid composition also contributes to flavour development, as shown in Cambodian *toeuk trey*, where raw fish lipid content was associated with the characteristics of the final product (*18*). Thus, fish sauce quality emerges from the interaction of microbial activity, enzymatic hydrolysis, metabolite formation, lipid transformation and physical breakdown of fish tissues (*19*).

Advances in high-throughput sequencing and metabolomics have improved understanding of fish sauce fermentation as a dynamic microbial–biochemical system. For example, *Halanaerobium*, a genus associated with spoilage-related metabolites such as butyrate, acetate and methylamine, was prevalent during the early stages of Chinese fish sauce fermentation with 30 % salt (*20*). Over time, *Halanaerobium* declined, *Halomonas* became dominant, and *Tetragenococcus* remained scarce due to salt sensitivity. Broader analyses of Chinese *yu-lu* consistently show the dominance of Firmicutes and Proteobacteria (*21*–*23*). Despite these advances, understanding of microbial succession across diverse fish species, salinity, production environments and regional fermentation systems remains limited, indicating a clear knowledge gap.

Although regional fish sauces differ in raw materials, fermentation methods, fish-to-salt ratios, and final product attributes (Table 2 (*13*, *18*, *24*-*32*)), they share a common principle: the gradual conversion of fish tissue into a flavour-rich liquid through autolytic and microbial transformations in saline environments (*26*). The degree of hydrolysis, fermentation time, microbial community structure and post-fermentation treatments determine differences in taste, aroma, colour, texture, nutritional quality and safety (*33*).

**Table 2:** Varieties of fish sauce from Asian countries

Fish sauce name	Country	Species	Appearance	*ξ*_fish,salt_	*w*(NaCl)/%	Fermentation time and condition	Ref.
*Budu*	Malaysia	anchovy, sardines, small herring	turbid	2:1 or 3:1	-	6–12 months	(*13*)
*Nam pla*	Thailand	anchovy	translucent	2:1 to 5:1	-	12–18 months fermentation, 2–4 weeks for ripening under the sun	(*24*, *25*)
*Nuoc mam*	Vietnam	anchovy	translucent	2:1 to 6:1	-	9–12 months or more of fermentation in the vats with recirculation	(*26*)
*Yu-lu*	China	anchovy	translucent	-	30–40	12–18 months fermentation, 1–2 weeks ripening in warm temperature (40–50 °C)	(*27*)
*Kecap ikan*	Indonesia	anchovy, small marine fish	translucent	-	25–30	10–12 months of fermentation	(*28*)
*Ishiru/ Shottsuru*	Japan	sardines	translucent	3:1 to 5:1	-	1 to 2 years of fermentation	(*29*–*31*)
*Myeolchi aekjeot*	Korea	anchovy	translucent	-	25	outdoor fermentation for more than 6 months	(*32*)
*Teuk Trei*	Cambodia	anchovy, gourami, *Channidae*	translucent	3:1 or 4:1	-	6–12 months in concrete vats or jars under the sun	(*18*)

Traditional fermentation provides distinctive sensory diversity, but its long duration and inconsistent microbial control limit scalability and standardisation. Accelerated fermentation technologies, including starter cultures, backslopping, enzymatic hydrolysis, controlled salinity, temperature optimisation and emerging physical treatments, offer opportunities to reduce fermentation time and improve product consistency. However, acceleration cannot be evaluated solely by fermentation speed. It must also consider microbial stability, protein and lipid degradation, the development of desirable volatile compounds and the control of safety risks. Therefore, an integrated framework is required to understand how microbial, biochemical and physical transformations interact during accelerated fish sauce fermentation.

Unlike previous reviews that focus primarily on either microbial diversity or flavour chemistry, this review integrates microbial ecology, biochemical transformations, and physical changes into a unified framework for process optimisation. It further evaluates acceleration strategies, particularly starter cultures, backslopping, enzyme-assisted fermentation and process control, in relation to their effects on fermentation kinetics, quality development, safety and product standardisation. This integrated perspective provides a scientific basis for optimising fish sauce fermentation while preserving its characteristic sensory quality and cultural identity.

## PHYSICAL CHANGES DURING FISH SAUCE FERMENTATION

Fish muscle is a protein-rich biological matrix characterised by a high moisture content and a distinct structural organisation. The muscle tissue is organised into fibres grouped within segmented myotomes and supported by connective tissue networks (*34*). Fish muscle contains less intramuscular connective tissue than mammalian muscle, which contributes to its softer, more easily digestible texture.

Fish muscle proteins are classified into three major groups: sarcoplasmic, myofibrillar and stromal proteins, each with distinct solubility and functional properties (*34*, *35*). The predominance of myofibrillar proteins makes fish muscle a suitable substrate for progressive structural disintegration during fermentation. As the myofibrillar framework is destabilised, the muscle fibre structure loses compactness, allowing the tissue to soften and gradually release soluble nitrogenous components. In contrast, stromal proteins and collagen-rich connective tissue are more resistant and therefore contribute to the persistence of residual solid material during fermentation. This structural heterogeneity explains why fish muscle does not liquefy uniformly. Instead, it undergoes progressive softening, partial solubilisation and gradual separation into liquid and insoluble fractions. The extent and rate of these changes may vary among raw materials because fish muscle composition differs across species and is influenced by biological and environmental factors, including age, sex, environment, feed intake and season (*36*).

### Role of autolytic enzymes, microbial enzymes and salt

Degradation of fish muscle proteins is primarily driven by autolytic enzymes, microbial proteases and salt. Autolysis occurs immediately *post mortem*, when endogenous enzymes, previously regulated in live fish, begin breaking down muscle tissue (*12*). Cathepsins and other lysosomal proteases hydrolyse structural proteins, while lipases degrade lipids, producing fatty acids that contribute to pH reduction. Lactic acid is also produced from glycogen through *post-mortem* glycolysis, further contributing to the decrease in pH. The combined action of lactic acid and fatty acids disrupts lysosomal membranes, releasing hydrolytic enzymes that promote the formation of peptides, free amino acids (FAA) and free fatty acids (FFA) (*12*).

The inclusion of visceral organs in whole-fish fermentation enhances autolysis due to digestive enzymes such as serine proteases (trypsin and chymotrypsin) and aspartic proteases (pepsin), which hydrolyse stomach contents and migrate into muscle tissue. This enzymatic activity facilitates microbial invasion, whereby autochthonous microorganisms further accelerate protein breakdown.

Salt concentration plays a dual role: it extracts liquid from muscle tissue while creating a hypertonic environment that suppresses undesirable bacteria and favours halotolerant fermentative microbes. Salt can denature proteins, making them more susceptible to enzymatic hydrolysis, and promotes lipid oxidation, contributing to flavour and colour changes (*37*). However, excessively high salt concentrations may inhibit proteolytic enzymes, prolonging fermentation and reducing protein breakdown rates (*38*). Environmental factors such as sunlight exposure can partially offset this inhibition by increasing enzyme activity.

### Liquefaction of fish muscle

Fish muscle liquefaction is predominantly caused by enzymatic hydrolysis of myofibrillar proteins, which are highly soluble in salt, while stromal proteins remain partially intact (*11*). The rate of hydrolysis is influenced by substrate availability, enzyme activity, microbial populations and salt concentration.

Faisal *et al*. (*38*) observed that fish treated with 25 % NaCl underwent faster muscle breakdown than those treated with 30–35 % NaCl, with complete bone exposure occurring at 255 days *versus* 270 days, respectively (*38*). This highlights the inhibitory effect of high salt content on proteolysis, counterbalanced by the hypertonic liquid extraction effect, which facilitates enzyme access to substrates. Trichloroacetic acid (TCA)-soluble peptides, indicative of proteolysis, increase as fermentation progresses (*39*, *40*). Denaturation of sarcoplasmic and myofibrillar proteins accelerates the breakdown, as evidenced by the reduced intensity of myosin heavy chain and actin bands, and the appearance of lower-molecular-mass degradation products (Table 3 (*14*-*18*, *41*-*44*)).

**Table 3:** Chemical profiles of commercial fish sauce samples from different regions

Type of fish sauce	pH	*w*(NaCl)/%	*w*(moisture)/%	*γ*(total N)/(g/100 mL)	*w*(protein)/%	*w*(lipid)/%	TSS/°Brix	TVBN as *w*(N)/(mg /100 g)	*a*_w_	Ref.
Malaysian *budu*	5.17	20.72	66.92	-	11.39	-	-	-	0.84	(*41*)
Indonesian *kecap ikan*	5.30	26.66	71.87	-	10.6	-	-	-	0.81	(*17*)
Indonesian *bakasang*	5.9	17	66.3	-	14.54	1.41	-	-	-	(*15*)
Korean *myeolchi-aekjeot*	5.49	22.2	67.4	1.27	7.94	-	-	-	-	(*16*)
Korean *myeolchi-aekjeot*	5.78	23.78	-	1.24	7.75	-	-	206.38	-	(*42*)
Chinese *yu-lu*	6.15	22	66	1.49	9.31	-	-	-	-	(*16*)
Thai *nam pla*	5.42	27.0	62.86	-	10.85	-	-	-	0.79	(*17*)
Vietnamese *nuoc mam*	5.75	20.2	61.4	2.59	16.19	-	-	-	-	(*16*)
Japanese *shottsuru*	5.54	18	69.2	1.8	11.25	-	-	-	-	(*16*)
Iranian *mahyaveh*	6.24	11.7	-	-	-	-	-	-	-	(*14*)
Cambodian *toeuk trey*	5.83	23.1	61.5	2	12.5	3.6	-	-	-	(*18*)
Italian *colatura di alici*	5.57	25.97	59.99	-	10.6	-	34.5	-	-	(*43*, *44*)

TSS=total soluble solids, TVBN=total volatile basic nitrogen

Proteolysis and liquefaction are further modulated by microbial enzymes, including proteases, lipases, decarboxylases and amine oxidases. Microbial activity complements autolysis, breaking down structural proteins, generating flavour compounds and contributing to the characteristic liquid form of fish sauce. Collagen degradation by microbial collagenases, together with endogenous trypsin and chymotrypsin, results in texture softening and separation of connective tissue from muscle fibres (*44*). Oxidative modification of myofibrillar proteins also alters texture through cleavage, cross-linking and interactions with lipid oxidation products (*45*).

### Colour development during fish sauce fermentation

Colour is a critical indicator of fish sauce quality. Freshly fermented fish sauce has a straw yellow colour, which gradually darkens to amber or brown during prolonged fermentation (*38*). Lightness (L*) values decrease over time, reflecting progressive browning (*9*). Colour formation is influenced by lipid oxidation, Maillard reactions between amino acids and reducing sugars, and enzymatic browning mediated by polyphenol oxidase and tyrosine oxidation. Variations in lipid content between lean and fatty fish affect browning intensity, while oxidation products interact with Maillard products to produce complex colour and flavour compounds. These physical transformations are closely linked to underlying biochemical reactions, particularly protein hydrolysis and metabolite formation, which ultimately determine flavour development.

## FISH SAUCE FLAVOUR-CONTRIBUTING METABOLITES

Different volatile and non-volatile metabolites are produced during fish sauce fermentation through autolysis and microbial enzyme activity. Their formation is influenced by environmental factors such as temperature, salt concentration, oxygen and light. As fermentation progresses, these metabolites develop further. Although not all generated volatile compounds contribute to the flavour of the product, several play a key role. Research has been conducted on the flavours and volatile compounds in fish sauces, and most volatile compounds are determined using headspace techniques, such as solid phase microextraction (SPME) combined with gas chromatography-mass spectrometry (GC-MS) (*1*). Advanced analytical techniques including electronic nose, SPME-GC-MS and headspace gas chromatography-ion mobility spectrometry (HS-GC-IMS) have enabled detailed characterisation of volatile compounds and their dynamic changes during fermentation, providing deeper insights into flavour development mechanisms (*46*). These approaches allow the identification of key aroma-active compounds and their temporal evolution, which is essential for designing strategies to accelerate fermentation without compromising sensory quality. Table 4 (*1*, *32*, *43*, *47*-*53*) summarises the volatile compounds found in various types of fish sauce.

**Table 4:** Volatile and non-volatile metabolites in different fish sauces: formation mechanisms and flavour characteristics

Fish sauce	Metabolite type	Metabolite	Mechanism of formation	Flavour characteristics	Ref.
Korean sand lance fish sauce *(kanari- aekjeot*)	volatile	3-methylbutanoic acid, butanoic acid, acetic acid, 2,6-dimethylpyrazine, benzaldehyde, 1-butanol, 1-penten-3-ol, 3-methyl-1-butanol, 2-methylbutanal	deamination and decarboxylation of valine, PUFA oxidation, Strecker degradation of leucine, Maillard reaction	cheesy, fermented, sweaty, rancid butter, cheesy, nutty, almond, roasted	(*1*, *43*, *47*, *48*)
Malaysian *budu*	volatile	ethanol, 3-methyl-1-butanol, 2-methylbutanal, 3-(methylthio)-propanal, 3-methylbutanoic acid, benzaldehyde	PUFA degradation, Strecker degradation of methionine, deamination and decarboxylation of valine	wine, onion-like, cheesy, fermented, sweaty, almond, bitter	(*43*, *47*–*49*)
Thailand *nam pla*	volatile	1-butanol, 1-penten-3-ol, 3-methyl-1-butanol, 2-methyl-1-butanol, 2-butanone, TMA, 2,6-dimethylpyrazine, benzene, phenol, acetic acid, propanoic acid, butanoic acid, 3-methylbutanoic acid, dimethyl disulfide	PUFA oxidation, Strecker degradation, Maillard reaction, degradation of TMAO	nutty, roasted, cheesy, fishy, faecal, sulfury	(*43*, *48*)
Vietnam *nuoc mam*	volatile	3-methylbutanal, 3-methyl-1-butanol, 2-methyl-1-butanol, acetone, 2-pentanone, TMA, pyridine, 3-ethyl-2,5-dimethylpyrazine, benzaldehyde, phenylethyl alcohol, acetic acid, butanoic acid, 3-methylbutanoic acid, 2-methylbutanoic acid, pentanoic acid, 4-methyl pentanoic acid	deamination and decarboxylation of valine, PUFA oxidation, Maillard reaction, Strecker degradation	cheesy, fermented, sweaty, rancid butter, nutty, almond, roasted, bitter	(*43*, *47*, *48*, *50*)
Chinese fish sauce	volatile	1-penten-3-ol, 2-butanone, TMA, phenol, acetic acid, dimethyl sulfide, 4-methyl pentanoic acid, 2-methyl butanoic acid, butanoic acid, propanoic acid	PUFA oxidation, degradation of TMAO, Maillard reaction, Strecker degradation	rancid butter, fishy, nutty, roasted, almond	(*43*, *48*, *50*)
Chinese *yu lu*	volatile	3-methylthiopropanal, 3-methylbutanal, 1-octen-3-ol, 2-methylpropanal, ethyl acetate	Strecker degradation of methionine, linoleic acid oxidation, esterification	onion-like, mushroom-like, sweet, fruity	(*51*, *52*)
South Korean fish sauce	volatile	1-penten-3-ol, TMA, benzaldehyde, acetic acid, butanoic acid, propanoic acid, 3-methylbutanoic acid, 2-methylbutanoic acid	PUFA oxidation, degradation of TMAO, Strecker degradation, Maillard reaction	fishy, cheesy, nutty, almond, roasted	(*43*, *48*, *53*)
Japanese fish sauce	volatile	1-butanol, 1-penten-3-ol, 3-methyl-1-butanol, ethyl acetate, phenylethyl alcohol, 3-methylbutanoic acid	PUFA degradation, esterification, Strecker degradation, deamination and decarboxylation of valine	wine, sweet, fruity, nutty, cheesy, fermented, sweaty	(*47*, *48*, *53*)
Italian *colatura di alici*	volatile	2-methyl-butanoic acid, 3-methyl-butanoic acid, nonanal, 2-methyl-butanal, octanal	deamination and decarboxylation, Strecker degradation	cheesy, nutty, almond	(*43*, *47*)
–	non-volatile	glucose, acetate, inosine, betaine TMA and DMA, biogenic amines, glycerol, fatty acyl hydroperoxides, carbonyl species	glycogen hydrolysis, carbohydrates (glucose) or lipids (glycerol) fermentation, hydrolysis of ATP, choline oxidation, reduction and/or demethylation of TMAO, decarboxylation of amino acids and nitrogen compounds, lipid hydrolysis, degradation and oxidation of unsaturated fatty acids	not reported	(*32*, *52*)

TMA=trimethylamine, TMAO=trimethylamine N-oxide, DMA=dimethylamine, PUFA=polyunsaturated fatty acids, ATP=adenosine triphosphate

Table 4 shows that the most abundant volatile compounds in fish sauce are 3-methylbutanoic acid, acetic acid, benzaldehyde, 2,6-dimethylpyrazine and trimethylamine (TMA). Other volatile compounds include acids, carbonyls, nitrogenous compounds and sulfur-containing compounds, which are formed during fermentation and contribute to the distinctive flavour of fish sauce depending on their odour activity value. Thai premium fish sauce has an overall aroma strongly influenced by aldehydes, acids and sulfur-containing compounds, according to Lapsongphon *et al*. (*54*), with 3-methylbutanal, 3-(methylthio)propanal, methylpropanal, butanoic acid, methanethiol and dimethyl trisulfide exhibiting the highest odour activity values (OAVs) (*54*). According to Wichaphon *et al*. (*55*), the taste sensation of Thai fish sauce arises mainly from salty and umami flavours and ammoniacal, meaty, and cheesy odour attributes (*55*). The aroma resulting from the combination of diverse volatile metabolites is the key attribute reflecting the quality of fish sauce.

### Mechanism of flavour formation

Through various biochemical metabolic processes, microorganisms and enzymes degrade raw materials into primary metabolites such as amino acids, fatty acids and glucose (*56*). Microorganisms use these primary metabolites for energy production to support their growth and metabolism, and to produce volatile secondary metabolites that contribute to the flavour and aroma of fermented fish sauce (*57*). Table 4 shows the volatile metabolites prevalent in fish sauce, their mechanisms of formation and their flavour characteristics. The generation of volatile compounds in fish sauce can be attributed to amino acid, fatty acid and glucose metabolism. The mechanism of flavour formation based on the initial substrate is described below.

#### Amino acid metabolism

In fish sauce, branched-chain amino acids (BCAAs), aromatic amino acids and sulfur-containing amino acids are key compounds for flavour formation (*58*). BCAAs can react with reducing sugars in the Maillard reaction and contribute to the formation of furans and pyrazines with roasted and nutty notes. Transamination of BCAAs, like leucine, isoleucine and valine, produces branched-chain α-keto acids (BCKAs). This transamination reaction involves oxidative deamination of one amino acid followed by reductive amination of another amino acid and is known as Stickland degradation. Interconversion of certain amino acids can produce pyruvate, which is used for microbial energy production. When BCKAs are decarboxylated, α-keto acid intermediates are formed. The oxidation of these intermediates produces the corresponding branched-chain aldehydes, such as 3-methylbutanal (isovaleraldehyde), from leucine. The conversion of an α-amino acid into an aldehyde is also known as Strecker degradation. Other Strecker aldehydes include isobutyraldehyde, 2-methylbutanal and methional, which are formed from valine, isoleucine and methionine, respectively (*59*). The formation of methyl aldehydes is primarily driven by microbial metabolism, which breaks down proline, leucine and phenylalanine into 2-methylpropanal, 3-methylbutanal and phenylacetaldehyde, respectively (*51*). Microorganisms such as *Enterococcus* and *Lactobacillus* have been reported to possess aldehyde-generating enzymatic activity (*53*).

Aromatic amino acids such as phenylalanine, tyrosine and tryptophan are decarboxylated to their corresponding aromatic amines, phenethylamine, tyramine and tryptamine, respectively. The acetyl group (-COCH_3_) from acetyl-CoA is then transferred to these aromatic amines by aromatic acetyltransferase to form acetate esters. Ethyl acetate is one of the esters in fish sauce that contribute to its fruity aroma. Cysteine and methionine are sulfur-containing amino acids that can be metabolised *via* various chemical reactions during fish sauce fermentation, which include removal of the sulfur atom, transamination and decarboxylation. Methionine-derived compounds, such as 3(methylthio)-propanal, contribute to onion-like odour. In addition to aldehydes and esters, amino acid derivatives can also further degrade to carboxylic acids and higher alcohols (*58*). The latter compounds are important immediate precursors of flavour-active esters (*1*).

The formation of higher alcohols from amino acids is known as the Ehrlich pathway. Phenylethyl alcohol and 2-methyl-1-propanol (isobutyl alcohol) are generated *via* phenylalanine and valine catabolism, respectively (*51*). In the Ehrlich pathway, an amino acid is decarboxylated to form an aldehyde and then reduced to form higher alcohols. Phenylalanine, for example, is decarboxylated to phenylacetaldehyde and then reduced to phenylethyl alcohol. Through esterification or alcoholysis, some yeasts can convert higher alcohols into volatile esters (*45*).

#### Fatty acid metabolism

Anchovies, the primary raw material used in fish sauce production, are rich in mono- and polyunsaturated fatty acids, which play a significant role in flavour development during fermentation. The autoxidation of these fatty acids, initiated either by physical catalysts such as light or by endogenous and microbial enzymes, is reported to be responsible for various flavour metabolites. Lipoxygenase (LOX) is the enzyme that catalyses polyunsaturated fatty acid (PUFA) oxidation, releasing hydroperoxides and volatile compounds. The fatty acid hydroperoxides are further cleaved by hydroperoxide lyase (HPL). The involvement of these enzymes in fatty acid metabolism can generate aldehydes, esters, branched-chain aliphatic alcohols and ketones (*60*). Among the oxidation products of oleic acid are nonanal and 2,4-heptadienal, which belong to saturated and unsaturated aldehydes. Hexanal is mainly derived from linoleic acid, an omega-6 unsaturated fatty acid (*61*). As lipid oxidative degradation products, volatile aldehydes contribute to desirable aromas (malt, grass, cheese and fruit) as well as rancid flavour (*62*). Aldehydes are often regarded as key flavour intermediates because they can undergo both oxidation and reduction to form the corresponding acids and alcohols (*43*).

The LOX system and β-oxidation pathways contribute to ester formation through the metabolism of fatty acids. The oxidation of α-linolenic acid, which is the key step in aroma ester synthesis, is regulated by the LOX gene. In the presence of unsaturated fatty acids and high LOX activity, the levels of ethyl hexanoate and hexyl acetate increase, indicating that these compounds are produced through fatty acid metabolism (*60*). Oxidative cleavage of higher-chain PUFA, α-linolenic acid, by LOX and HPL releases the unsaturated C6 volatiles such as hexanoic acid and hexanal. Hexanal provides a green grass aroma, while hexanoic acid contributes fatty and pungent notes (*60*). It has also been noted that oxidation of linoleic acid or arachidonic acid results in the formation of 1-octen-3-ol in fish sauce (*63*). The study conducted by Ding *et al.* (*64*) showed that octanal and nonanal in fish sauce are highly correlated with oleic acid content, while 1-octen-3-ol is highly correlated with linoleic acid.

#### Glucose metabolism

Carbohydrates in fish sauce are primarily glycogen. Glycogenolysis breaks down glycogen and forms glucose-6-phosphate. Through glycolysis (Embden-Meyerhof-Parnas pathway), the key flavour intermediate, pyruvate, is formed from glucose-6-phosphate with the assistance of three key enzymes: phosphofructokinase, hexokinase and pyruvate kinase (*65*). Oxidation and deamination of some amino acids, such as alanine, can also result in pyruvate. The entry of this compound into the tricarboxylic acid cycle can lead to the formation of various metabolic intermediates. The beta-oxidation of fatty acids can yield acetyl-CoA, which can enter the tricarboxylic acid cycle and be converted to various compounds, including pyruvate. Table 4 lists different types of non-volatile compounds in various types of fish.

Furans and pyrazines are two groups of volatile compounds formed through reactions involving reducing sugars, primarily glucose, and amino acids. An increase in temperature during fish sauce fermentation may promote the formation of furans *via* Amadori rearrangement pathways (*47*). This process begins with a reaction between the carbonyl group of glucose and the amino group of amino acids, forming an Amadori compound (Schiff base intermediate). Under elevated temperature or low pH conditions, the carbon backbone of the Amadori compound undergoes rearrangement, leading to the formation of furan ring structures. Pyrazines, in contrast, are mainly formed through Maillard reactions. Amadori intermediates can undergo further transformations, resulting in the formation of nitrogen-containing heterocyclic compounds, which are characteristic of pyrazines.

### Role of microbes in flavour formation

Flavour in fish sauce develops through a dynamic and complex biochemical process influenced by microbial composition, metabolic activity and fermentation conditions. As fermentation progresses, volatile compounds are continuously produced, particularly during maturation, when interactions among protein, lipid and carbohydrate derivatives generate increasingly complex flavour profiles. Flavour, which arises from the combination of taste and aroma, is a key determinant of consumer acceptance of fermented fish products. Consequently, the microbial community plays a critical role in shaping both flavour and overall sensory characteristics (*63*). Several studies have demonstrated correlations between microbial composition and flavour development, as well as microbial succession during fermentation (*51*, *53*, *66*, *67*). Changes in bacterial communities over time are influenced by variations in chemical composition and fermentation parameters, including acid accumulation, salt concentration and temperature. The fermentation of most fish sauces occurs at high salt concentrations, a condition that favours the growth of halophilic or halotolerant microorganisms.

In a study by Udomsil *et al*. (*68*), *Staphylococcus* sp. CMC5-3-1 modulated the umami attribute of fish sauce as shown by the highest content of glutamic acid and proline in fish sauce inoculated with this strain (*68*). In another study, staphylococci were reported to possess alcohol and aldehyde dehydrogenase activities that contribute to ethanol oxidation. Ethanol is first converted into acetaldehyde and then oxidised into acetic acid, contributing to flavour development in fish sauce (*50*). *Halanaerobium* sp. is reported to produce butyrate, acetate, propionate and biogenic amines through the fermentation of monosaccharides, amino acids and glycerol (*67*). A 180-day fermentation after the addition of lactic acid bacteria (LAB) strains, for example *T. halophilus*, to anchovy digested with protease (0.25 % Alcalase and 0.5 % Flavourzyme) showed an improvement in the amino acid content in the resulting fish sauce, particularly glutamic acid, leucine, tyrosine and phenylalanine. LAB have been reported to have high intracellular aminopeptidase activity towards glutamic acid, which may contribute to elevated glutamic acid concentrations in *T. halophilus*-inoculated samples (*68*).

Leucine, isoleucine and valine (BCAAs) are important precursors of branched aldehydes such as 2-methylpropanal, 2-methylbutanal and 3-methylbutanal. The elevated concentrations of leucine and valine observed in *T. halophilus*-inoculated samples may contribute to the formation of volatile aldehydes associated with fish sauce flavour development. The addition of *T. halophilus* eliminates the undesirable dimethyl disulfide formed by transamination of methionine, which contributes to a faecal note. In another study, inoculation with *Virgibacillus* sp. SK37 led to an increase in volatile aldehydes such as 2-methylpropanal and 2-methylbutanal (*68*). The involvement of *Tetragenococcus* in carbohydrate metabolism in Chinese fish sauce has been reported (*66*). Malic and succinic acids are produced through carbohydrate metabolism, specifically through the citrate cycle. *Tetragenococcus* is also involved in the metabolism of arachidonic acid and methionine, contributing to mushroom-like odour and roasted potato-like odour, respectively. Other findings in Chinese fish sauce highlighted the importance of this species, along with *Halanaerobium, Halococcus, Halomonas* and *Candidatus frackibacter*, in fish sauce fermentation and the synthesis of flavour compounds (*51*).

Understanding these metabolic pathways is essential for accelerating fermentation, as targeted manipulation of key substrates and microbial activity can promote faster formation of desirable flavour compounds while minimising off-flavours.

## MICROBIAL COMMUNITY PROFILE OF FISH SAUCE

A wide variety of microbial communities develop during fish sauce fermentation, but certain types are more common. Halophilic and proteolytic bacteria are the key microorganisms in fish fermentations, which gradually degrade protein along with fish endogenous enzymes. The following section presents the methods used to identify microorganisms involved in fish sauce fermentation.

### Methods used for microbial community analysis

Although the microbial community in naturally fermented fish sauce is complex, understanding it can substantially deepen insight into the factors that determine its safety and quality. The contribution of microorganisms can be evaluated by correlating biogenic amine (BA) profiles with microbial community composition derived from sequencing data (*66*). Both culture-dependent and culture-independent techniques have been used to characterise the microbial community in fish sauce.

#### Plate-dependent technique

Culture-dependent techniques involve growing and enumerating microorganisms on media with specific nutrient compositions. In this approach, fish sauce samples are diluted and transferred into liquid media or spread onto agar plates, which provide a favourable environment for specific microbial species to grow. After incubation, visible colonies that arise from individual microbial cells can be counted, isolated and further analysed as part of phenotypic identification. The presence of diverse bacteria during the fermentation of Malaysian fish sauce has been studied using this technique (*13*, *69*). These studies have shed light on some of the viable microbial species. Halophilic LAB, such as *Lactococcus* and *Lactobacillus*, have been identified along with *Micrococcus*, *Staphylococcus, Pediococcus, Candida* and *Saccharomyces* using a culture-dependent method (*13*). *Staphylococcus, Bacillus* and *Micrococcus* were found to be predominant microorganisms during fermentation of fish sauce (*70*). Additionally, *Enterobacteriaceae*, *Micrococci* and *Lactobacilli* have also been reported in fish sauce (*71*).

However, the complex microbial consortia that are likely to be involved in fermentation remain unknown because of several study limitations. Culture-based techniques have low sensitivity and tend to underestimate true microbial diversity because most viable bacteria cannot be grown under stress, and viable but non-cultivable cells are difficult to assess (*72*). Many microbial species have unique growth requirements or dependencies that are difficult to replicate under artificial laboratory conditions. To date, various bacteria, including *Achromobacter, Bacillus, Halomonas, Micrococcus, Brevibacterium, Halobacterium, Vibrio, Flavobacterium, Staphylococcus* and *Tetragenococcus* species, have been identified in fish sauces mainly by culture-dependent approaches despite the presence of many uncultivable microbes (*57*).

#### Molecular technique

Various plate-independent techniques, also known as molecular or genome-based identification methods, have been developed for bacterial community analysis. These techniques allow the direct study of microbial communities from environmental samples without the need for traditional cultivation. Deoxyribonucleic acid (DNA)-based methods are widely used to overcome the limitations of phenotypic identification, particularly the underestimation of microbial diversity in fermented foods. A culture-independent method called polymerase chain reaction–denaturing gradient gel electrophoresis (PCR-DGGE) has been used to study bacterial diversity in *ngari*, an Indian fermented seafood (*62*) and in Japanese *ajinarezushi* and *iwashi-nukazuke* (*73*). Fukui *et al*. (*74*) used molecular cloning to reveal bacterial diversity in a fish sauce mash during fermentation, together with cultivation studies to better understand the fish sauce microbiota (*75*).

With the advent of next-generation sequencing (NGS) technologies, metagenomics and high-throughput sequencing are increasingly useful for identifying functional genes specific to microbial communities and their metabolic networks (*75*). NGS can provide quantitative information on the abundance of microorganisms and help address the shortcomings of the previously mentioned techniques, particularly their low resolution. A study conducted by Subasinghe *et al*. (*76*) showed that PCR-DGGE and clonal sequencing were able to identify 20 bacterial genera in all enriched and non-enriched media. In comparison, NGS was able to identify 114 bacterial families and 134 genera based on V3 and V6 regions, demonstrating its superiority in revealing genetic diversity.

Ohshima *et al.* (*67*) applied 16S rRNA amplicon next-generation sequencing to investigate bacterial communities during industrial Thai fish sauce fermentation. In this culture-independent approach, microbial DNA was extracted from fish sauce mash samples, the bacterial 16S rRNA gene region was amplified, and the resulting amplicons were sequenced using a high-throughput sequencing platform. The generated sequence reads were then processed to profile bacterial taxa and compare community dynamics across fermentation stages and manufacturing plants. This approach allowed a broader characterisation of microbial succession than culture-dependent methods, particularly for detecting uncultivable or low-abundance bacteria in high-salt fermentation environments.

The importance of data preprocessing and quality control has been described by He *et al*. (*77*). The obtained high-quality clean data were then subjected to *de novo* assembly. In this step, the reads are computationally assembled into long contiguous sequences (contigs) to reconstruct microbial genomes or genes. Alternatively, reads can be mapped to reference genomes or gene databases for alignment and identification. Taxonomic classification and functional annotation were performed using the processed reads or the assembled contigs. They were compared to databases such as the National Center for Biotechnology Information (NCBI) GenBank (*78*) or specialised databases to identify and classify microbial taxa and predict functional genes and pathways. Various statistical and data visualisation techniques were used to interpret the results, assess diversity, identify differentially abundant taxa or genes, and investigate associations and correlations.

Application of NGS in fish sauce microbiome analysis revealed the distinct diversity of fish sauces produced by different companies, suggesting significant effects of raw materials, fermentation vessels and environment on bacterial diversity. In a comparison between two companies, *Halanaerobium* sp. was the initially dominant genus in one company, gradually replaced by *Lentibacillus* sp., *Halomonas* sp. and *Tetragenococcus* sp., which appeared in the middle phase of fermentation. In the other company, the most common genera were *Peptostreptococcus* sp., *Peptoniphilus* sp., *Gallicola* sp., *Fusobacterium* sp., *Halanaerobium* sp. and *Vagococcus* sp. (*67*).

#### Microbial community changes during fermentation

Advances in sequencing technology have shed light on the fish sauce microbiome and how the structure of the community changes over time. Table 5 (*21*, *23*, *32*, *67*, *79*) shows different types of microorganisms identified at different fermentation stages. The composition and succession of microorganisms vary throughout fermentation due to changes in the fermentation matrix. During the 12-month fermentation of Chinese fish sauce prepared with 30 % salt, the microbial community changes. A dominant presence of *Halanaerobium*, a microorganism known to indicate spoilage and excessive generation of butyrate, acetate and methylamine in fermented fish products, was observed (*21*). As fermentation progressed, the bacterial community became more diverse, and the relative abundance of *Halanaerobium* rapidly decreased after 6 months. The *Halomonas* genus emerged as one of the detectable genera after 6 months of fermentation, and by 12 months, it had become the dominant group (*23*).

**Table 5:** The microbial community in fish sauces at different stages of fermentation

		Dominant microbe			Ref.
Fish sauce	Fermentation condition	Early stage	Middle stage	Late stage
*Myeolchi-aekjeot* (Korea)	*m*(short-length anchovy): *m*(solar salt):*m*(brine), *w*(NaCl)=25 %)=10:2.7:4, fermentation was carried out at 25 °C for 280 days	Gammaproteobacteria, *Salinivibrio, Staphylococcus, Bacillus, Photobacterium, Tetragenococcus*	*Tetragenococcus, Staphylococcus, Halanaerobium*	*Tetragenococcus, Halanaerobium*	(*32*)
*Yu lu* (China)	Anchovies mixed with 30 % salt and fermented for 12 months	*Halanaerobium*, *Fusobacterium, Photobacterium*	*Halanaerobium*, *Halanaerobacter*	*Halanaerobium, Halomonas*	(*23*)
Traditional Chinese fish sauce (TCFS)	*m*(anchovy):*m*(salt):*m*(brine)=21:8:30 (brine: *w*(NaCl)=30 %), fermentation for 15 months	*Shewanella*	*Lactococcus, Bacillus, Halanaerobium*	*Tetragenococcus, Pseudomonas, Psychrobacter, Tissierella, Carnobacterium,* and *Gallicola*	(*21*)
Thai fish sauce	*m*(fish):*m*(salt)=10:3, fermented in cement fermentation tanks in open area for 15 months	*Halanaerobium*	*Halanaerobium, Lentibacillus, Halomonas, Tetragenococcus*	*Lentibacillus, Tetragenococcus, Halococcus, Halomonas, Alkalibacillus*	(*67*)
Fujian fish sauce	*m*(anchovy): *m*(water):*m*(salt)=10:0.8:1	*Carnobacterium*	*Tetragenococcus*	*Peptostreptococcus, Lentibacillus*	(*79*)

For instance, a study on Malaysian budu revealed a distinct succession in which the microbiota shifted from being dominated by *Halanaerobium* and *Staphylococcus* at 6 months to *Lentibacillus*, *Bacillus* and *Halomonas* at 12 months of fermentation (*80*). This microbial succession correlated with a shift in metabolic pathways from degradation to biosynthesis of protein and lipid derivatives, leading to the accumulation of key flavour compounds that indicate sauce maturity and quality (*80*). In the same sauce, *Tetragenococcus* was detected at low concentrations, suggesting that the high-salt environment does not support this LAB species (*23*).

Most studies on the fish sauce microbiome have reported the predominance of Firmicutes and Proteobacteria, including in Chinese yu-lu (*21*-*23*). In some fish sauces, Firmicutes dominated during the early fermentation period, followed by Proteobacteria at the end (*40*). However, other studies found that Proteobacteria dominated at the beginning of fermentation but were rapidly replaced by Firmicutes towards the end (*20*, *21*, *32*). The dominance of Firmicutes throughout the entire fermentation process has been reported in Thai fish sauce (*67*) and Fujian fish sauce (*79*).

These insights into microbial succession provide a basis for accelerating fermentation through the early establishment of functional microbial communities using starter cultures or controlled inoculation strategies.

## ATTEMPTS TO ACCELERATE FERMENTATION AND ENHANCE FLAVOUR FORMATION

During fermentation, autolytic enzymes and halophilic microorganisms gradually degrade proteins and lipids, producing peptides, free amino acids, fatty acids and volatile compounds responsible for the characteristic flavour (*58*,*81**,**82*). Flavour-forming microorganisms are not dominant at the early stage; they are progressively selected and established as fermentation proceeds (*83*). This slow microbial succession explains why high-quality fish sauce often requires more than one year to mature. Consequently, omics approaches such as metagenomics, metabolomics and volatilomics are increasingly important for identifying key microbes, metabolic pathways and flavour-related compounds, enabling rational starter culture selection to accelerate fermentation while maintaining product quality.

## LEVERAGING MULTI-OMICS APPROACHES FOR STARTER CULTURE SELECTION

Recent advances in high-throughput and data-driven technologies offer new opportunities to accelerate and standardise fish sauce fermentation. Omics-based approaches, including metagenomics, metatranscriptomics, metabolomics and volatilomics, enable comprehensive characterisation of microbial communities, functional genes, metabolic pathways and flavour-related compounds involved in fermentation. These tools can identify key microorganisms associated with rapid proteolysis, amino acid formation, volatile compound production and desirable sensory attributes, thereby supporting the rational selection of starter cultures with targeted functional traits.

Several studies have demonstrated the value of integrating microbial community analysis with metabolomic and volatilomic profiling in fermented fish systems. In Malaysian fish sauce, Lestari *et al.* (*80*) linked bacterial community structure and predicted metabolic activities with volatile compound formation, showing that *Halanaerobium, Staphylococcus, Halomonas*, *Bacillus* and *Lentibacillus* were associated with maturity-related volatile compounds. Similarly, metagenomic analysis has revealed characteristic flavour metabolic networks in fish sauce microbiota under different fermentation processes (*50*), while comparative analysis of fast-fermented Chinese fish sauces has shown strong associations between microbial succession and volatile flavour formation (*53*). Beyond fish sauce, Zhao *et al.* (*82*) demonstrated that high-throughput sequencing, volatile analysis and metabolomics can clarify the relationship between microorganisms and flavour development in dry-cured grass carp, supporting the broader application of multi-omics approaches in fermented fish products. These findings indicate that integrating omics data with systems biology and data-driven modelling may help design optimised microbial consortia and controlled fermentation strategies.

## USE OF STARTER CULTURE

Fish sauce is typically fermented without starter cultures and relies on native bacteria derived from the raw materials. Owing to the complexity of these native strains, product quality is not always homogeneous and the fermentation duration can vary (*84*). Consequently, the use of starter cultures has been explored not only as a quality assurance strategy, but also as a means to accelerate fermentation and guide flavour formation (*85*). The selection of starter cultures is particularly important because strains not derived from fish sauce may fail to adapt to high-salt, protein-rich fermentation conditions or compete effectively with the native microbiota. In some cases, non-indigenous starters may also alter the characteristic sensory profile of traditional fish sauce. Therefore, autochthonous microorganisms isolated from fish sauce or related fermented fish products are considered more suitable candidates, as they are already adapted to high salinity, complex substrates and microbial competition within the fish sauce ecosystem (*86*).

Recent studies have demonstrated that reconstructing microbial communities through cooperative starter cultures can improve both the quality and safety of low-salt fish sauce by enhancing functional microbial interactions and reducing undesirable metabolites (*87*). Importantly, such approaches may also help shorten fermentation time by promoting the early establishment of proteolytic and flavour-producing microbiota, enhancing early-stage proteolysis and guiding flavour development pathways.

Supporting this approach, halotolerant *Bacillus* strains isolated from Malaysian budu have shown proteolytic, lipolytic, esterolytic and glutamic acid-accumulating activities, indicating their potential roles in protein hydrolysis, lipid transformation, aroma formation and umami development (*81*). These strains have been proposed as a cocktail culture to accelerate fermentation and enhance the flavour profile of fish sauces, leveraging their well-adapted nature (*81*).

Several studies have investigated the application of starter cultures in fish sauce fermentation and their effects on microbial succession, flavour formation and product quality, as summarised in Table 6 (*22,**68*, *83*, *84*, *88*-*93*). Various strains, including *Bacillus*, *Tetragenococcus*, *Halanaerobium*, *Virgibacillus*, *Staphylococcus*, *Marinococcus*, *Planococcus* and *Psychrobacter*, have been characterised for their proteolytic activity and salt tolerance and applied in laboratory-scale fermentation either as single-strain or mixed-strain cultures at inoculation typically ranging from 10^6^– to 10^7^ CFU/g. These starter cultures may also produce enzymes that enhance flavour development or inhibit undesirable metabolites such as histamine and dimethyl trisulfide (*68*, *85*).

**Table 6:** Starter culture strains used in fish sauce fermentation and their impact on fish sauce microbiota and quality

Fish sauce production	Strain added	Impact on microbiota	Impact on fish sauce quality	Ref.
Minced whole fish+18 % NaCl, 35 °C, stirring conducted every 5 days until 45 days	*Bacillus subtilis* B-2 (10^6^ CFU/g)	Lower microbial community richness and evenness possibly due to inhibition of spoilage microorganisms. *Bacillus* remained prominent during entire period of fermentation	Aldehydes with pleasant grass, fat and flower notes were enhanced. Higher 1-octen-3-ol (mushroom flavour) and 1-penten-3-ol (roasted onion flavour	(*88*)
Minced scad meat+18 % NaCl, 35 °C, 45 days	*Tetragenococcus muriaticus* YL9-5 (10^6^ CFU/g)	*Tetragenococcus* become the main bacteria in fish sauce. they clustered with *Synechococcus, Rhodococcus, Stenotrophomonas, Achromobacter* and *Brucella*	Hexanal, nonanal and benzaldehydes ranked highest among volatile compounds followed by esters	(*22*)
2 kg salted anchovy, 30 °C, 6 months	100 mL *Marinococcus halotolerans* SPQ culture+500 mL fish exudate (10^6^–10^7^ CFU/g)	The count of inoculated strains decreased over time and reached zero after 6 months of fermentation	Inoculated fish sauce had a better aroma and a higher glutamic acid content than control	(*84*)
Anchovy+25 % sea salt 150 days	*Virgibacillus halodenitrificans* PS21 and *Staphylococcus simulans* PMRS35 (10^6^ CFU/g)	Until day 150, proteolytic bacteria increased in inoculated samples. Those inoculated with *V. halodenitrificans* PS21 have a faster increase in proteolytic bacteria	DH, FFAs, thiobarbituric acid reactive substances (TBARS), total amino acid profiles, browning intensity aldehydes, ketones, alcohols, hydrocarbons, furans and FFAs were lowest in non-inoculated samples. Inoculated samples had lower contents of dimethyl disulfide, which contributed to faecal notes	(*89*)
Minced scad+20 % NaCl, 35 °C, 45 days	*Halanaerobium fermentans* YL9-2 (10^6^ CFU/g)	Not studied	Significant increase in AN and aldehyde concentrations	(*83*)
Minced longsnout catfish, prehydrolysis with dispase (55 °C, 2 h) and papain (45 °C, 3 h), 8 % salt, 15 days	*Planococcus maritimus* XJ2 (10^6^–10^7^ CFU/g)	Not studied	Inoculated samples had higher AN and lower TVBN. Higher concentration of compounds contributing to a malt and nutty aroma (hexanal, 2-methylbutyraldehyde and 3-methylbutyraldehyde)	(*90*)
Anchovy+25 % salt, 180 days	Sequential inoculation of *Virgibacillus* sp. SK37 and *T. halophilus* MS33	All inoculated samples' bacterial counts decreased gradually over 180 days of fermentation	Inoculated samples had high AN and DH, higher propanal, 2-methylbutanal, methylpyrazine and lower dimethyl disulfide concentrations than the uninoculated ones	(*91*)
Anchovy+0.25 % Alcalase (65 °C, 2 h)+0.5 % Flavourzyme (50 °C, 4 h), 25 % salt, 35 °C	10 % (*V*/*m*) *Staphylococcus* sp. CMC5-3-1 (10^5^-10^6^ CFU/mL)	Bacterial counts of fish sauce decreased as fermentation progressed	Higher concentrations of glutamic acid, proline and 2-methylpropanal than the control, α-amino group content and butanone comparable to the control	(*68*)
Half fin anchovy+15 % salt, 30 °C 120 days continued by 50 °C 10 days	*Psychrobacter* sp. SP-1 (10^7^ CFU/g)	Moderately halophilic microbial counts increased following inoculation	Inoculation enhanced protease activity, AN, total soluble nitrogen (TSN), umami taste and meaty aroma	(*92*)
Anchovy+0.25 % Alcalase (60 °C, 2 h)+0.5 % Flavourzyme (50 °C, 4 h), 25 % salt, 35 °C	*Virgibacillus* sp. SK33, *Virgibacillus* sp. SK37 and *Staphylococcus* sp. SK1-1-5	No increase in numbers was observed in inoculated cultures. As fermentation progressed, fish sauce bacteria count decreased	Treatment with proteinases and starter culture could produce fish sauce with comparable sensory qualities within four months	(*93*)

AN=amino nitrogen, DH=degree of hydrolysis, FFA=free fatty acids, TBARS=thiobarbituric acid reactive substances, TSN=total soluble nitrogen, TVBN=total volatile basic nitrogen

The primary objective of using starter cultures is to enhance proteolytic activity. Key quality attributes of fish sauce, such as amino nitrogen (AN), taste-active peptides and amino acid derivatives, are closely associated with protease activity and contribute significantly to the final flavour profile. These attributes can be developed more rapidly by incorporating proteases or microbial strains with high proteolytic activity (*81*, *82*). The relationship between protease activity and taste compound formation has been investigated. Changes in protease activity were found to correlate with the formation of free amino acids, AN, TCA-soluble peptides and organic acids during the fermentation of anchovy sauce, while changes in 5′-nucleotide content did not correlate with protease activity. According to variable importance for projection analysis, alanine, glutamic acid, lysine, aspartic acid, leucine, TCA-soluble peptides and succinic acid are key variables that indicate changes in protease activity during anchovy sauce fermentation (*61*).

### Backslopping

Backslopping is a traditional fermentation method in which a small portion of a finished ferment is added to a fresh substrate as an inoculum. This inoculum carries an established microbial community and metabolites that may help initiate fermentation, shorten the microbial adaptation phase and promote the early dominance of microorganisms responsible for desirable flavour, aroma, texture and safety attributes. In spontaneously fermented foods, backslopping is commonly used to stabilise fermentation performance and maintain product identity across batches, as reported for sourdough, fermented dairy products and cereal–legume fermentations (*94*–*97*).

In fermented aquatic products, backslopping remains less explored, but available evidence suggests that it is a promising approach. In Thai fermented fish (*plaa-som*), backslopping was compared with spontaneous fermentation and mixed-strain inoculation using lactic acid bacteria isolated from plaa*-*som. Although the inoculated treatment showed higher LAB counts, the backslopped samples had lower pH and reduced Enterobacteriaceae counts, while sensory attributes such as colour, aroma, taste, sourness, texture and overall acceptance were not significantly different among treatments (*98*). This suggests that backslopping may improve fermentation control and microbial safety without compromising traditional sensory characteristics.

Although direct studies on backslopping in fish sauce remain limited, the approach is scientifically plausible because mature fish sauce or budu contains salt-adapted microorganisms and flavour-related metabolites formed during the later stages of fermentation. Adding mature broth or a small portion of a high-quality previous batch may increase the initial population of functional halophilic or halotolerant microorganisms, promote earlier proteolysis and amino acid formation, and help preserve the region-specific taste and aroma of traditional fish sauce. However, the quality and safety of the previous batch are critical, since backslopping may also transfer undesirable microorganisms or biogenic amine-producing bacteria. Therefore, backslopping for fish sauce should be combined with microbial profiling, metabolite analysis and biogenic amine monitoring before broader industrial application.

## USE OF ENZYME-ASSISTED AND LOW-SALT STRATEGIES

Enzyme-assisted fermentation has been widely explored as an effective approach to accelerate fish sauce production by enhancing protein hydrolysis and increasing the release of peptides, free amino acids and flavour precursors. Adding commercial proteases drastically reduces fermentation time compared to traditional spontaneous methods, which often require over a year to achieve adequate proteolysis and flavour depth. Alcalase and Flavourzyme have been used in research, with trials demonstrating that enzyme-aided Thai fish sauce ripens in just four months, matching the amino acid content of 12-month commercial benchmarks (*93*). Protex 51FP has similarly enhanced peptide and amino acid yields in 180-day anchovy fish sauce fermentation (*99*).

Recent approaches have further combined enzymatic hydrolysis with low-salt fermentation and selected microbial starter cultures to improve both fermentation efficiency and flavour development. A recent study combined neutral protease and Flavourzyme with a microbial blend of *Lactiplantibacillus plantarum*, *Zygosaccharomyces rouxii*, *Aspergillus oryzae* and *Bacillus subtilis* to accelerate low-salt tilapia fish sauce production while enriching volatile aromas and metabolites (*100*). Such integrated strategies are particularly promising because enzyme treatment alone may accelerate hydrolysis but does not necessarily reproduce the characteristic flavour complexity of traditional fish sauce. The incorporation of functional microorganisms can therefore complement enzymatic digestion through amino acid metabolism, ester formation, lipid transformation and aroma biosynthesis. Moreover, low-salt fermentation systems may further enhance microbial and enzymatic activity, although careful microbial control is required to maintain safety and prevent spoilage or excessive biogenic amine formation.

Salt reduction can also accelerate fermentation by increasing enzyme activity and microbial metabolism. In naturally fermented fish sauce, endogenous proteases, including cathepsins, contribute to protein degradation, particularly when pH changes promote enzyme activity (*68*, *100*). Lowering the salt concentration, in combination with starter cultures, has been shown to increase protein solubilisation during fish sauce fermentation (*93*). Proteolytic *Bacillus* starter cultures have also been reported to improve the overall taste of fish sauce, likely through enhanced protein breakdown and flavour precursor formation (*88*). However, low-salt and enzyme-assisted systems present important technological and safety challenges. Reduced salt concentrations may increase microbial and enzymatic activity, but they can also permit the growth of spoilage or biogenic amine-producing microorganisms if fermentation is not properly controlled. Future development should therefore focus on optimising enzyme dosage, salt concentration, starter culture performance and safety monitoring to achieve rapid fermentation without compromising flavour authenticity, product stability or consumer safety.

Taken together, acceleration strategies for fish sauce fermentation should be viewed as process-control interventions. Starter cultures, backslopping, enzyme-assisted hydrolysis, low-salt systems and omics-guided microbial selection can accelerate proteolysis, amino acid release and flavour precursor formation; however, their success depends on maintaining the balance among fermentation kinetics, microbial ecology, biochemical maturation, product safety and sensory authenticity. Integrating these emerging technologies with traditional practices has the potential to improve process efficiency, reduce fermentation time and ensure consistent product quality, although their application in fish sauce systems remains limited and warrants further investigation.

Despite significant advances, several limitations remain. The lack of standardised microbial consortia and limited understanding of functional interactions among halophilic species constrain the reproducibility of accelerated fermentation systems. Additionally, most studies are conducted at laboratory scale, with limited validation under industrial conditions. Future development should therefore prioritise integrated multi-omics analysis, kinetic modelling, targeted starter design, sensory validation and safety monitoring to establish accelerated fermentation systems that are rapid, reproducible, safe and sensorially comparable to traditionally matured fish sauce.

## CONCLUSIONS

Fish sauce fermentation involves interconnected microbial, enzymatic, biochemical and physical processes that shape its quality, safety and distinctive sensory profile. While traditional spontaneous fermentation can generate complex and characteristic flavours, it is time-consuming and often produces inconsistent outcomes. Strategies such as starter culture application, backslopping, enzyme-assisted hydrolysis, salt reduction and combined enzyme-microbial systems show potential to accelerate protein breakdown, amino acid formation and flavour development. At the same time, multi-omics tools can clarify microbial-metabolic relationships and guide more precise starter selection and process control. Further studies are still needed to confirm their safety, scalability, biogenic amine control and ability to retain authentic flavour. Therefore, efforts to accelerate fish sauce fermentation should aim not only to reduce processing time, but also to control the key transformations required to produce fish sauce that is safe, consistent, efficient and comparable to traditionally fermented products.
